# Morphometric study of the suprascapular notch and scapular dimensions in adult Malawian cadavers and implications of completely ossified superior transverse scapular ligament

**DOI:** 10.11604/pamj.2022.41.324.33205

**Published:** 2022-04-21

**Authors:** Thom Kaledzera, Brian Matundu, Gbenga Anthony Adefolaju, Juziel Manda, Anthony Mwakikunga

**Affiliations:** 1 Biomedical Sciences Department, Anatomy Division, School of Life Sciences and Allied Health Professions, Kamuzu University of Health Sciences, Blantyre, Malawi,; 2 Physiotherapy Department, School of Life Sciences and Allied Health Professions, Kamuzu University of Health Sciences, Blantyre, Malawi,; 3 School of Medicine, University of Central Lancashire, Preston, United Kingdom

**Keywords:** Morphology, morphometry, suprascapular notch, suprascapular neuropathy, scapula

## Abstract

**Introduction:**

the anatomy of the suprascapular notch and its relationship to scapular dimensions are critical in the management of suprascapular neuropathies. Individuals show considerable differences in the dimensions of the suprascapular notch across populations. The purpose of this study was to determine the morphology and morphometric dimensions of the suprascapular notch in adult Malawian cadavers and to suggest clinical implications associated with complete ossification of the suprascapular ligament.

**Methods:**

adult dry scapulae from undetermined sex specimens (n=125) obtained from the skeletal collection at Kamuzu University of Health Sciences were classified according to the Rengachary categorization method to assess the suprascapular notch superior transverse distance, mid transverse distance, depth, scapula length and width using a standard Vernier caliper.

**Results:**

the most prevalent suprascapular notch class was type I, which was found in 46 (36.8%) of all scapulae. Type VI was the least common, found in only 1 (0.8%) of the scapulae. The mean notch superior transverse distance was 1.3 ± 0.6 cm, while the mean maximum depth was 0.6 ± 0.3 cm. Only the differences in depth, however, were statistically significant (p=0.001).

**Conclusion:**

the current study has described the morphology and morphometry of the suprascapular notch in relation to the risk of suprascapular nerve entrapment associated with complete ossification of the suprascapular ligament. Our sample population generally showed smaller suprascapular notch and scapular dimensions than other populations. This should be considered during the management of suprascapular neuropathy and preoperative planning of surgical operations of the shoulder region.

## Introduction

Knowledge of anatomical variations and anomalies along the course of the suprascapular nerve is important in the diagnosis, prevention, and assessment of suprascapular neuropathies including bony and brachial plexus injuries. This knowledge is also important for surgeons carrying out surgical interventions at the shoulder region. The superior border of the scapula is the shortest of the three borders, with the suprascapular notch directly medial to the root of the coracoid process. The suprascapular nerve passes beneath the superior transverse scapular ligament and inferior transverse scapular ligament at the fibro-osseous canals of the suprascapular notch and spinoglenoid notch, respectively [1]. The suprascapular nerve originates at the base of the neck from the superior trunk of the brachial plexus. It runs posterolaterally from the origin, through the suprascapular foramen where it innervates the supraspinatus muscle. It then passes through the spinoglenoid foramen, formed by the root of the spine of the scapula, before terminating and innervating the infraspinatus muscle. Along its course, the nerve also provides branches that innervate the shoulder joint and the acromioclavicular joint [1,2]. Complete ossification of the superior transverse scapular ligament with formation of bony foramina is the most recognised predisposing factor for compression at the suprascapular notch [3,4]; it is also a risk factor in surgical exploration for suprascapular nerve decompression [5]. Compression or entrapment of the nerve underneath the suprascapular ligament often leads to a suprascapular neuropathy, which clinically manifests with pain around the shoulder region, weakness and atrophy of the supraspinatus and infraspinatus muscles [6]. Suprascapular neuropathy affects 1-2% of all patients who present with shoulder region pain [7].

Rengachary *et al*. in 1979, proposed a classification system for the human suprascapular notch based on its anatomy. The suprascapular notch could be classified as type I, type II, type III, type IV, type V and type VI. Type I scapulae have a large depression across the length of the scapula's superior border, from the medial superior angle to the base of the coracoid process. Type II has a wide V-shaped notch that is blunted, but type III has a symmetrically U-shaped notch with parallel margins. A small V-shaped narrow notch distinguishes type IV. Types V and VI are similar to type III except for partial or total ossification of the suprascapular ligament [8]. One of the most common risk factors for developing suprascapular neuropathy is a narrow suprascapular notch, particularly of type VI based on a Reganchary classification system [3,9]. Existing evidence from literature, however, suggests that the percentage of suprascapular notches with type VI differs by population, ranging from as low as 0.7% to as high as 9.7% in some areas [3,4,9-12].

The distribution of suprascapular notch Rengachary types and their relationships with anthropometric dimensions of the entire scapula has been extensively investigated in numerous populations, including Uganda, Kenya, the United States and India [3,9-11,13,14]. The incidence of complete ossification of the suprascapular ligament has received a lot of attention. Individuals show considerable differences in the asymmetric dimensions of the suprascapular notch and suprascapular ligament across populations. The purpose of this study was to determine the morphology of the suprascapular notch and morphometric dimensions of the scapular in adult Malawian cadavers and to suggest clinical implications associated with complete ossification of the suprascapular ligament.

## Methods

The current study was conducted in accordance with the Government of Malawi, Anatomy Act No. 14 of 1990 (updated 2016) and was approved by the Kamuzu University of Health Sciences, Formerly University of Malawi´s College of Medicine Research and Ethics Committee (COMREC) with a clearance number P02/10/872. The study was conducted on adult human cadaveric dry scapulae obtained from the Anatomy Division´s Bone Collection housed in the Biomedical Sciences Department, School of Life Sciences and Health Professions, Kamuzu University of Health Sciences. The study sample included only the scapulae prepared from adult Malawian cadavers. A total of 125 undamaged and pathology free scapulae obtained from individuals of undetermined sex aged between 21-82 years were selected for the study.

**Exclusion criteria:** the study excluded all scapula bones with a broken area along their superior border or at the suprascapular notch that were sufficient enough to affect the measurements or classification.

**Figure 1:**
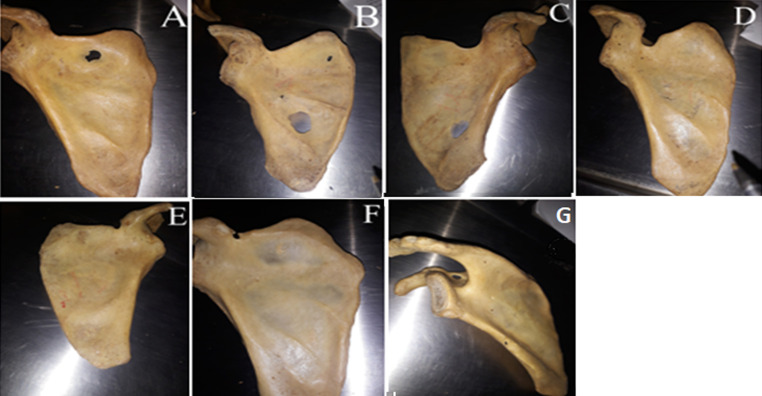
photographs showing the various morphological classification of the suprascapular notch: A) absent; B) type I; C) type II; D) type III; E) type IV; F) type V; G) type VI

**Figure 2:**
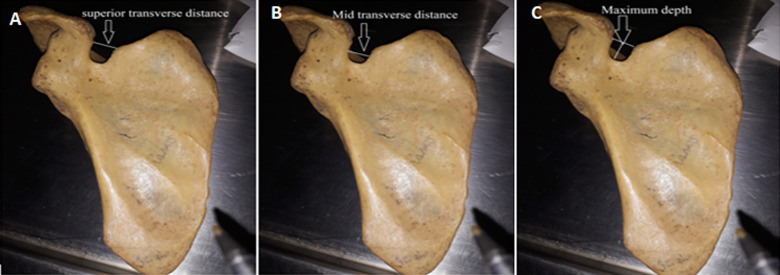
morphometric measurements of the suprascapular notch to assess: A) the superior transverse distance; B) mid transverse distance; C) maximum depth

**Figure 3:**
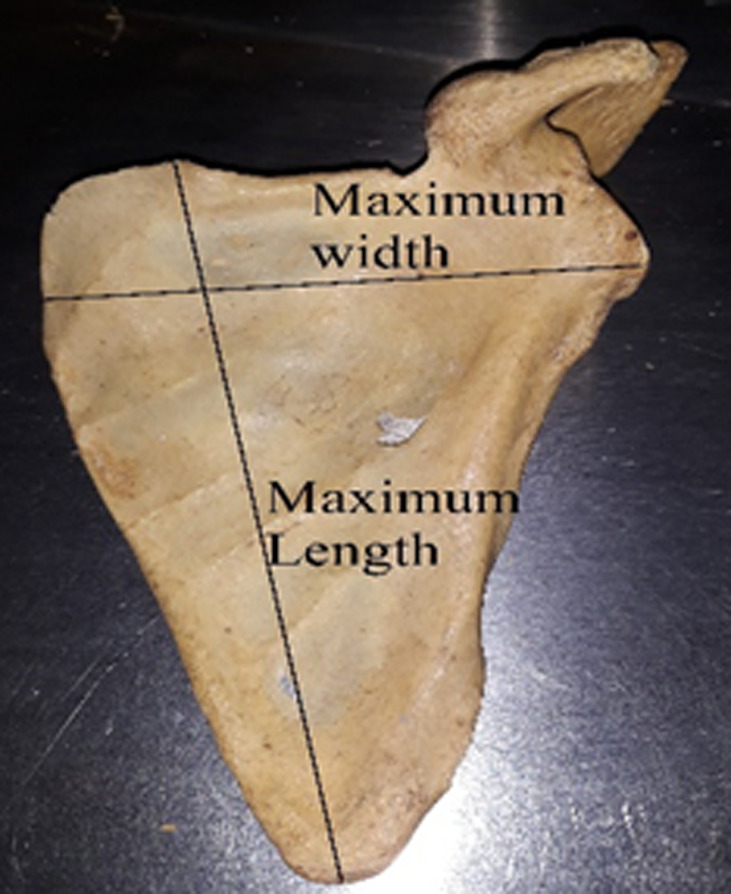
anthropometric measurements of the scapula; measurements of the maximum length and maximum width of the scapula

**Data collection method and tools used:** to morphologically group suprascapular notches, a Rengachary classification system was used. Type I, II, III, IV, V, or VI notches were assigned (Figure 1). Each notch was assigned a class on the basis of an agreement among all investigators present at the time of data collection. When researchers noticed an unusual morphology in a bone, they made a note of it by describing how it appeared. The representative photographs for all morphological kinds were taken with a digital camera. Two investigators independently took all of the metric measures using a standard Vernier caliper. Only until they agreed on the precise measurement of a particular bone was the data recorded on the data collection sheet. The following measurements were taken (Figure 2 and Figure 3): 1) superior transverse distance - measured between the superior ends of the area at the suprascapular notch; 2) mid transverse distance - a transverse distance measured halfway through the suprascapular notch; 3) maximum depth - the distance measured between the superior transverse line and the deepest point of the notch; 4) maximum length of the scapula; 5) maximum width of the scapula.

**Data management and analysis:** the IBM SPSS Statistics 28.0.0.0 computer software was used to analyze the data. Frequencies, means, standard deviations and percentages were used to provide descriptive data in tables. To determine the significance of the differences found, the mean distance differences between various types were compared using one way ANOVA followed by a Tukey Kramer post hoc analysis. A p-value <0.05 was considered significant at the 95% confidence interval. The maximum transverse distance of the suprascapular notch was compared with the maximum width of the scapula using a bivariate Pearson correlation. Likewise the maximum depth of the suprascapular notch was compared with the maximum length of the entire scapula. Findings from this study were then compared with results from similar studies done in other populations.

## Results

**Figure 4:**
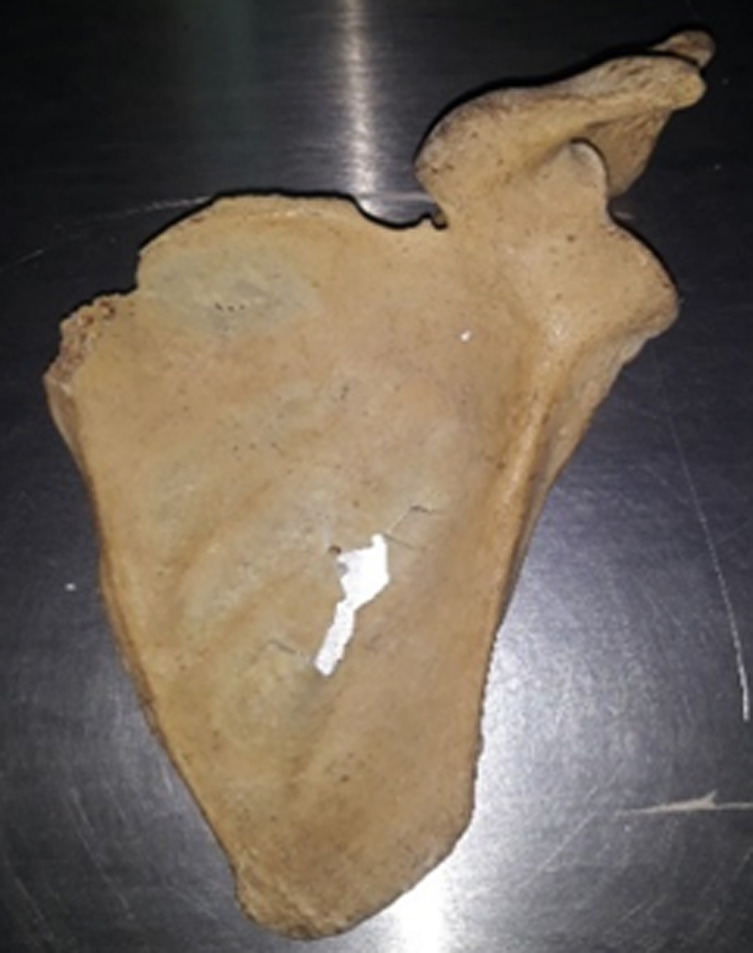
unusual morphology of the suprascapular notch; not fitting in either type of the rengachary classification system

A total of 125 scapulae were examined, with 66 (52.8%) belonging to the right upper limb and 59 (47.2%) belonging to the left upper limb. The suprascapular notch was present in 120 (95%) of the scapulae (Table 1). The most common form of the suprascapular notch was type I, which was found in 46 scapulae (36.8%). The least common type was type VI, which was found in only one case (0.8%). The distribution of the scapular types is shown in Table 1. Out of the 125 scapulae which were analyzed, 1 (0.8%) had an unusual morphology that could not be classified into either type of the Rengachary classification system (Figure 4).

**Table 1:** morphological classification of suprascapular notch with frequencies and percentages

	Classification of the notch							Total
	Absent	Type 1	Type 2	Type 3	Type 4	Type 5	Type 6	
Frequency	6	46	21	37	5	9	1	125
Percentage	4.8	36.8	16.8	29.6	4.0	7.2	0.8	100

The mean superior transverse distance was 1.3±0.5cm, with variations depending on the notch type. It was found to be the greatest in type III, measuring 1.4±0.5cm, and the smallest in type IV, at 0.7±0.1cm (Table 2). However, these differences were not statistically significant (p-value= 0.980). The maximum depth for the suprascapular notch had a mean distance value of 0.6±0.3cm. Type III suprascapular notches had the maximum mean depth of 0.8±0.2cm and the least mean depth was observed in type IV, which was 0.4±0.1cm. These differences, however, were statistically significant (p-value= 0.001). On average, the scapula measured 14.6±0.8cm in length and 10.03±0.8cm in width. Type IV had the greatest length but the smallest width of all the types, measuring 15.4±1.2cm in length and 10.0cm in width. The mean differences in scapula lengths and widths did not differ significantly (p-values=0.444 and 0.864 respectively). The width of the scapula had a weak positive correlation with the suprascapular notch's superior transverse distance, which was however not statistically significant (r=0.105, p-value=0.595). The relationship between the maximal depth of the suprascapular notch and the length of the scapula was also not statistically significant (r=-0.070, p-value=0.724).

**Table 2:** a summary of descriptive analyses and comparison of means of the suprascapular notch superior transverse distance, mid transverse distance, maximum depth, maximum length and maximum width between suprascapular notch morphological classes

Variable	Superior transverse distance (cm)			Mid transverse distance (cm)			Maximum depth (cm)			Maximum length (cm)			Maximum width (cm)		
	II	III	IV	II	III	IV	II	III	IV	II	III	IV	II	III	IV
Mean	1.313	1.433	0.650	1.013	1.172	0.400	0.375	0.772	0.350	14.550	14.328	15.350	10.313	10.183	10.000
Standard deviation		0.4728	0.0707	0.7680	0.3409	0.1414	0.2605	0.2445	0.0707	0.7946	1.1726	1.2021	0.5592	0.8913	0.0000
Standard error	0.2689	0.1114	0.0500	0.2715	0.0804	0.1000	0.0921	0.0576	0.0500	0.2809	0.2764	0.8500	0.1977	0.2101	0.0000
P-value	0.980			0.840			0.001*			0.444				0.864	
Remark	NS			NS			S			NS			NS		

n= 125; S= Significant difference; NS= No significant difference

## Discussion

The current study examined the morphology and morphometric dimensions of the suprascapular notch in adult Malawian cadavers and their clinical implications associated with complete ossification of the suprascapular ligament. Numerous studies reported on the variations of the morphology and morphometric dimensions of the suprascapular notch in various populations [11,13,14]. These dimensional variations are population specific and may be attributed to genetic, environmental and regional differences [9-11]. Clinically, such variations have profound implications in the management of patients with symptoms associated with suprascapular neuropathy particularly suprascapular nerve entrapment associated with complete ossification of the suprascapular ligament [13-15].

Type I suprascapular notch morphology was the most common class reported in this study, with 46 (36.8%) of all scapulae studied falling into this category. In the Ranketi *et al*., 2010 study, 40 scapulae (29%) were type III, which was the commonest [3]. Meanwhile, in this study, 37 (29.6%) scapulae were found to be type III, and it was not the commonest, which is close, despite the sample analysed being less in the current study. The prevalence of type I in the present study, however, is consistent with the findings from a similar study by Kumar *et al*. 2014 in Singapore, who reported 87 scapulae (32.46%) [15]. On the contrary, type II suprascapular notch was the most common type in India observed in 61 scapulae (34.65%) and in China observed in 171 scapulae (58.16%). Type IV was the most common type in Italy which was observed in 156 scapulae (31.1%) [5,16,17]. This suggests the interpretation that the current observation of the variant suprascapular notch morphology supports population specificity on this anatomical feature.

In the current study, the complete ossification of the suprascapular notch was observed in 1 scapulae (0.8%). When compared to the incidences reported in other parts of Africa by Ranketi *et al*. 2010 in Kenya who reported 4 scapulae (2.9%) and Adewale *et al*. 2020 in Uganda who reported 10 scapulae (8%), the incidence rate of the current study is generally smaller [3,11]. However, the rate of complete ossification of the suprascapular ligament in this study was higher than that reported by Natsis *et al*. 2007 and Polguj *et al*. 2013 [13,18]. This means that complete ossification of the suprascapular notch appears to be population specific.

Notably, the suprascapular notch size in the present study was generally greater in type III and smaller in type IV in all measurements taken. Except for the maximum depth, the variations were not statistically significant. These findings agree with studies which were done in India [12,19,20]. Taken together, the metric values and morphology of the suprascapular notch have clinical implications in the entrapment of the nerve and are associated with the pathogenesis of suprascapular neuropathy. Therefore, findings of the present study imply that, although type VI is associated with a high risk of suprascapular nerve entrapment, it may further enhance the risk of the condition due to its small size in this study population suggesting that this should be considered during the management of suprascapular neuropathy and preoperative planning of surgical operations of the shoulder region.

**Strength and limitation of the study:** this research was done on unpaired scapulae bones of unknown sex. Therefore, the morphological and morphometric features observed could not be related to sex or allow a bilateral comparison. However, the methods used provided an assessment of the morphology of the suprascapular notch and scapular dimensions, with the risks of suprascapular nerve entrapment in cadaveric adult Malawians. It lays the groundwork for future radiological research to better link suprascapular neuropathy symptoms to the morphology and morphometry of the notch in living people.

## Conclusion

The current study has described the morphology and morphometry of the suprascapular notch in relation to the risk of entrapment of the suprascapular nerve associated with complete ossification of the suprascapular ligament, which seem to be population specific. This should be considered during the management of suprascapular neuropathy and preoperative planning of surgical operations of the shoulder region. Such an anatomical suprascapular notch description will also clinically help in identifying those who are at risk of developing suprascapular neuropathy.

### What is known about this topic

- The suprascapular notch could be classified into 6 main categories based on its morphology;
- A complete ossification of the suprascapular ligament, leading to a type VI suprascapular notch, is the most recognized predisposing factor to a nerve compression;
- The occurrence of this complete ossification of the suprascapular ligament varies by population.

### What this study adds

- The occurrence of complete ossification of suprascapular ligament in a Malawian population is at 0.8%;
- Our sample population generally shows smaller suprascapular notch and scapular dimensions than other populations;
- A weak and positive correlation exists between the measurements of the scapula and those of the suprascapular notch. However, the relationship lacks statistical significance.
